# Post COVID-19 Vaccine Related Cerebral Venous Sinus Thrombosis and Thrombocytopenia

**DOI:** 10.7759/cureus.20932

**Published:** 2022-01-04

**Authors:** William Butler- Manuel, Usman Iqbal Rana, Mansoor Zafar, Azeem Gadi, Amarah Kiani

**Affiliations:** 1 Internal Medicine, Conquest Hospital - East Sussex Healthcare NHS Trust, St. Leonards-on-Sea, GBR; 2 Gastroenterology and Hepatology, Conquest Hospital - East Sussex Healthcare NHS Trust, St. Leonards-on-Sea, GBR; 3 Emergency Medicine • Accident and Emergency, Conquest Hospital - East Sussex Healthcare NHS Trust, St. Leonards-on-Sea, GBR; 4 Radiology, Conquest Hospital - East Sussex Healthcare NHS Trust, St. Leonards-on-Sea, GBR

**Keywords:** vaccine-induced prothrombotic immune thrombocytopenia (vipit), thrombosis with thrombocytopenia syndrome (tts), cerebral venous sinus thrombosis (cvst), vaccine-induced immune thrombocytopenia and thrombosis (vitt), covid 19 vaccination, covid 19

## Abstract

During the height of the COVID-19 pandemic, there was great relief with the global mass rollout of the Covid-19 vaccination programs. While they have proven to be safe and effective, the gradual emergence of side effects to the vaccines has undermined public trust in the vaccination program and, whilst rare, can lead to significant morbidity and mortality. The most serious was the emergence of vaccine-induced immune thrombocytopenia and thrombosis (VITT), also known as thrombosis with thrombocytopenia syndrome (TTS) or vaccine-induced prothrombotic immune thrombocytopenia (VIPIT). VITT is a serious and often fatal complication of some COVID vaccines that seem more prevalent in younger people and women. We present a case of a 48-year-old woman who presented with VITT following COVID vaccination.

## Introduction

An unusual syndrome of thrombosis and thrombocytopenia was first noticed in late February 2021 in a small number of individuals who received the ChAdOx1 CoV-19 vaccine (AstraZeneca, University of Oxford, and Serum Institute of India). Similar findings were observed in a small number of individuals who received the Ad26.COV2. S vaccine (Janssen; Johnson & Johnson) both being adenoviral vector-based vaccines [1]. All the patients involved were tested negative for SARS-CoV-2 infection, but soon the link with recent vaccination became apparent. The exact incidence of vaccine-induced immune thrombocytopenia and thrombosis (VITT) remains unknown; however, reports have described only a small number of cases among tens of millions of vaccinated individuals. Despite being of rare occurrence, it is associated with high mortality, so clinicians must consider VITT in patients presenting with a petechial rash or headache post-vaccination. Surprisingly most cases are reported in younger people and females, as in our patients. We hope it may increase awareness of its existence as many cases may have been unnoticed and unreported.

## Case presentation

A 48-year-old female presented to the emergency department (ED) with a 6-day history of generalized fatigue and worsening severity of headache. She had received the Astra Zeneca COVID-19 vaccine 11 days previously. She had a past medical history of left eye amblyopia, and her sister had suffered a thrombotic stroke at 47 years of age. On examination, she was found to have a generalized petechial rash on her arms, legs, and face and cold sores in the mouth. Her Glasgow coma scale (GCS) was 15/15 with no focal neurological deficit. Initial blood tests showed: haemoglobin 118 (125-165), white cell count (WCC) 4.4 X 109/ l, platelets 11 x 109/l (150-400), international normalization ratio (INR) of 1.4 (0.8-1.2), prothrombin time (PT) 14.6 (10.0-11.7), activated partial thromboplastin ratio (APTR ratio) of 1.12 (0.85-1.10), fibrinogen level of 0.7 (1.8-3.6) and d-dimers of 10000 (- 225) (Table 1).

**Table 1:** Lab parameters ! by ELISA (King’s College London, Hospital)

Lab parameters tested	Units	Range	Day 1; 16:00 hrs	Day 1; 20:00 hrs	Day 2; 06:00 hrs	Day 2; 14:00 hrs
Hemoglobin (Hb)	g/L	125-165	118	108	106	104
White cell count (WCC)	x 10^9^/L	4-11	4.55	3.82	2.5	3.62
Platelets	x 10^9^/L	150-400	11	14	17	37
International normalisation ratio (INR)	-	0.8-1.2	1.4	1.5	1.2	1.4
Prothrombin time (PT)	seconds	10-11.7	14.6	15.0	12.9	14.2
Activated partial thromboplastin ratio (APTR)	-	0.85-1.10	1.12	1.11	1.21	1.6
Fibrinogen level	g/L	1.8-3.6	0.7	0.6	1.1	0.9
d-dimers	ng/ml	-225	>10000	>10000	>10000	>10000
Beta 2 microglobulin	mg/L	0-2.5		1.94		
PFA4 antibodies!	ng/ml	4-24	2.495			
Lactate dehydrogenase (LDH)	U/L	-250	-	313	-	-
Serum Total Creatinine Kinase	U/L	25-200		333		

**Figure 1:**
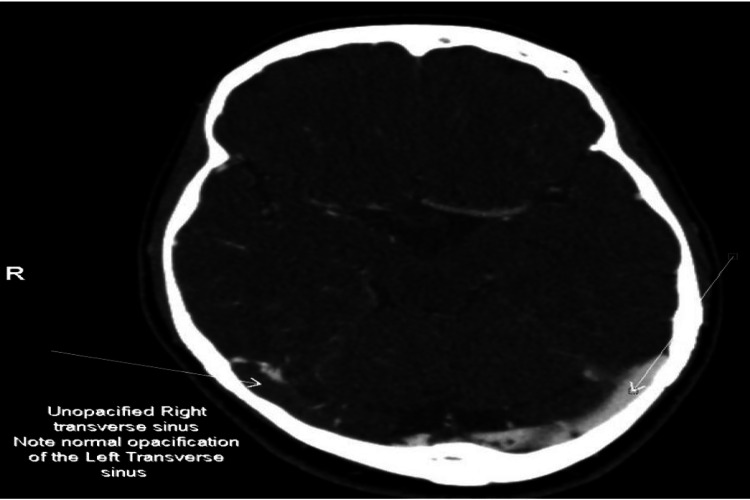
Axial view of the CT cerebral Venogram shows unopacified, thrombosed Right Transverse sinus.

**Figure 2:**
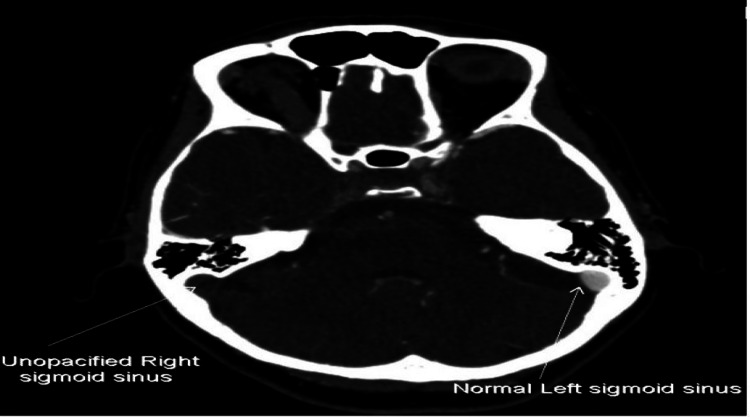
Axial view of the CT cerebral Venogram demonstrates non-enhancement of the thrombosed Right Sigmoid sinus.

Her blood film showed true thrombocytopenia with occasional large forms (megakaryocytes). No white cell abnormalities were demonstrated. She had a computerized tomogram (CT) head venogram scan showing thrombosis in the right transverse sinus and right sigmoid sinus on the right side. After discussing with the hematology team and considering the CT venogram and blood findings, she suspected that she had VITT (Figure 1 and 2). She was managed initially with 20 milligrams (mg) of dexamethasone followed by intravenous immunoglobulins (IVIG) 0.5g/kg daily for two days to reverse the autoimmune process. In consultation with the tertiary center, Argatroban was advised to be started only once the platelet count improved to more than 30 x 109/l. She was prescribed fibrinogen concentrate to achieve a target fibrinogen of 1.5, and PFA4 antibodies were sent. Additional blood tests include ADAMST13, direct antiglobulin test (DAT), antibodies, antiphospholipid screen (lupus anticoagulant, anti-cardiolipin antibody, anti-b2-glycoprotein antibody) paroxysmal nocturnal hemoglobinuria (PNH) screen, were requested, which were negative. The second day, following a multi-disciplinary meeting (MDM), she was advised to be transferred urgently to a tertiary neurosurgical center for plasma exchange & observation if she were to deteriorate.

Following transfer to the tertiary center, her GCS dropped. A repeat CT-head showed significant subarachnoid hemorrhage (SAH), with midline shift, raised intracranial pressure & early signs of coning. Plasma exchange was performed overnight without any clinical improvement, and platelet count continued to fall. Her condition deteriorated, and she was palliated and died two days later.

## Discussion

Since December 2020, the World Health Organisation (WHO) has issued various vaccinations emergency use listings (EUL). This includes Pfizer/BioNTech Comirnaty vaccine on December 31st, 2020, SII/Covishield and AstraZeneca/AZD1222 vaccines on February 16th, 2021, Janssen/Ad26.COV 2.S on 12 March 2021, Moderna COVID-19 vaccine (mRNA 1273) on 30 April 2021 and the Sinopharm COVID-19 vaccine on 7 May 2021 and Sinovac-CoronaVac was listed for EUL on June 1st, 2021 [2]. Globally, as of 4:50 pm CET, November 19th, 2021, there have been 255,324,963 confirmed cases of COVID-19, including 5,127,696 deaths, reported to WHO. As of November 18th, 2021, a total of 7,370,902,499 vaccine doses have been administered [3]. The WHO recommended that the vaccine rollout be fair and equitable worldwide to ensure that the most vulnerable groups are reached as soon as possible [4].

Much like initial uncertainty over the exact symptoms and associations of Covid-19 infection [5-7], several side effects of the vaccines were initially stipulated, most commonly skin rashes [8]. Soon, however, a few cases of VITT began to emerge as a rare but yet devastating side-effect of the adenoviral vector COVID-19 vaccines. In the United Kingdom (UK), the rapid, mass rollout of the vaccination program has led to several cases of VITT. Sue Pavord et al., in a prospective cohort study, evaluated 294 patients and identified 170 definite and 50 probable cases of VITT. The time from vaccination to presentation ranged from 5 to 48 days, with a median presentation 14 days after vaccination. The age range varied from 18 to 79 years with a median age of 48 years, with no sex preponderance and no specific medical risk factors. They observed mortality was 73% among patients with platelet counts below 30,000 per cubic millimeter and associated intracranial hemorrhage. They have concluded that the approximate incidence of VITT was at least 1:100,000 among patients 50 years of age or older and at least 1:50,000 among patients in the younger group [9]. Studies have suggested VITT appears far more likely following AstraZeneca/Johnson and Johnson adenoviral vaccines than Moderna/Pfizer mRNA vaccines [9].

Recommended treatment options include systemic glucocorticoids, including intravenous methylprednisolone, oral or intravenous dexamethasone, and oral prednisolone. Patients with a baseline platelet count of less than 30,000 per cubic millimeter were more likely to receive a platelet transfusion, typically in preparation for neurosurgery or anticoagulation. Anticoagulation was administered in 91% of the patients; non-heparin-based anticoagulation, including dabigatran, fondaparinux, apixaban, and argatroban, was used in 68%. Heparin was administered during admission to the hospital in 50 patients (23%). Its use was universal in the patients who presented before mid-March and received a diagnosis retrospectively. It was administered to other patients before the diagnosis was considered, and the anticoagulant was switched. Mortality among these patients was 20%, compared with 16% among those receiving non-heparin-based anticoagulants. Interventional radiologists performed neurosurgery or thrombectomy among patients with extensive cerebral venous sinus thrombosis with or without secondary intracranial hemorrhage. Lastly, along with anticoagulation, intravenous immunoglobulin has been an important treatment modality for the antibodies; although, patients with more severe disease may have benefitted from plasma exchange [10].

The case we present here is similar to other reported case findings of thrombosis at unusual sites, thrombocytopenia, disproportionately elevated d-dimer levels, and reduced fibrinogen levels [10-12]. VITT is associated with high titers of antibodies to platelet factor 4 (PF4) that activates platelets. It is an unusual condition similar to heparin-induced thrombocytopenia, a prothrombotic condition where the same antibodies are seen after heparin exposure. It has been suggested that patients who present with thrombosis and a normal platelet count post-vaccination might be in an early stage of VITT [12-15].Anti-SARS-CoV-2 monoclonal antibodies (mAbs) have been shown to have a clinical benefit in treating SARS-CoV-2 infection. They have also been found to be effective in preventing SARS-CoV-2 infection in household contacts of infected patients [15] and during SARS-CoV-2 outbreaks in nursing and assisted living facilities [16]. 

This raises the clinical dilemma, that if the patients with a history of coagulopathy choose to decline vaccination or are deemed high risk with some Covid-19 vaccinations, could they also be considered for the monoclonal antibody treatment by AstraZeneca, as approved by The Food and Drug Administration (FDA) the USA for emergency use on December 8th, 2021, for long-term prevention of COVID-19 among people with weakened immune systems before they have been exposed to the coronavirus with six-monthly injections [17]. Future data collection and analysis would enable us to see the same benefits for immunocompromised patients and patients with a history of coagulopathy. This consequentially may have a favorable outcome, unlike our patient. 

Why VITT appears to be more prevalent in younger age groups and females remains a mystery. However, it has been suggested that more young female care workers were vaccinated initially [18]. It is also debatable if patients with a personal or family history of prothrombotic states should be managed differently, as most patients with VITT did not have pre-existing clotting problems. In our patient with a family history of thrombotic stroke at a younger age, it is unclear if she was at higher risk of VITT and maybe should not have been eligible for vaccination. This suggests that their use can help similarly limit the spread of COVID to vaccination and may be an alternative if vaccination is thought to be too risky in some individuals. 

## Conclusions

The case emphasizes the need for more awareness and understanding of VITT, particularly the younger population. We have shared our experience hoping that future cases will have a more favorable outcome. More data collection and analyses are needed to establish if there are risk factors for VITT that should be considered before offering an adenoviral vector-based vaccine to young people.

## Appendices

Dr. William Butler-Manuel, Dr. Usman Iqbal Rana and Dr. Mansoor Zafar are joint first co-authors.
